# Supplementary material for: Fractionation and Extraction Optimization of Potentially Valuable Compounds and Their Profiling in Six Varieties of Two Nicotiana Species
Source: Molecules. 2022 Nov 21;27(22):8105. doi: 10.3390/molecules27228105 (PMC9694777; doi:10.3390/molecules27228105)
Supplement: Supplementary file 1 [file molecules-27-08105-s001.zip › molecules-2021398-supplementary.pdf]

Table S1. Extraction method summary.

Method No.	Extraction Solvents	Base Extraction Methods
M1	CH_2Cl_2	BM1
M2	CH_2Cl_2	BM2
M3	CH_2Cl_2/MeOH (4:1, *v/v*)	BM1
M4	CH_2Cl_2/MeOH (4:1, *v/v*)	BM2
M5	CH_2Cl_2/MeOH (1:1, *v/v*)	BM1
M6	CH_2Cl_2/MeOH (1:1, *v/v*)	BM2
M7	CH_2Cl_2/MeOH (1:4, *v/v*)	BM1
M8	CH_2Cl_2/MeOH (1:4, *v/v*)	BM2
M9	MeOH	BM1
M10	MeOH	BM2
M11	MeOH/H_2O (9:1, *v/v*)	BM1
M12	MeOH/H_2O (9:1, *v/v*)	BM2
M13	MeOH/H_2O (7:3, *v/v*)	BM3

Table S2. List of analytes and internal standards, and their selected properties: ESI polarity, monoisotopic mass (*m/z*), and retention time (RT).

No.	Compounds	ESI	*m/z*	RT
1	Nicotine	Pos	163.12297	4.60
2	Nicotine-*d4*	Pos	167.14809	4.60
3	Anatabine	Pos	161.10732	3.96
4	Anatabine-*d4*	Pos	165.13244	3.96
5	Anabasine	Pos	163.12297	3.70
6	Anabasine-*d4*	Pos	167.14809	3.70
7	Myosmine	Pos	147.09167	4.38
8	Myosmine-*d4*	Pos	151.11679	4.37
9	Nicotinamide	Pos	123.05529	2.60
10	Nicotinamide-$^{13}C_6$	Pos	129.07542	2.60
11	Cotinine	Pos	177.10224	3.58
12	Cotinine-*d3*	Pos	180.12107	3.58
13	Nornicotine	Pos	149.10732	3.24
14	Nornicotine-*d4*	Pos	153.13244	3.23
15	Norcotinine	Pos	163.08659	3.35
16	Norcotinine-$^{13}C_3$	Pos	166.09666	3.35
17	Nicotinic acid	Pos	124.03931	1.63
18	Nicotinic acid-$^{13}C_6$	Pos	130.05943	1.63
19	Chlorogenic acid	Pos	355.10236	4.42
20	Cryptochlorogenic acid	Pos	355.10236	4.63
21	Neochlorogenic acid	Pos	355.10236	2.63
22	*cis*-Zeatin	Pos	220.11929	1.38
23	*trans*-Zeatin	Pos	220.11929	1.64
24	*trans*-Zeatin-*d5*	Pos	225.15067	1.62
25	Ferulic acid	Pos	195.06519	5.48
26	Isoferulic acid	Pos	195.06519	5.38
27	Rutin	Pos	611.16066	5.31
28	Rutin-*d3*	Pos	614.17949	5.31
29	Xylitol	Neg	151.06120	2.50
30	Xylitol-*d7*	Neg	158.10513	2.50
31	Ambroxide	Pos	237.22129	3.50
32	Linoleic acid	Pos	281.24751	4.55
33	Linoleic acid-$^{13}C_{18}$	Pos	299.30789	4.58
34	α-Tocopherol	Pos	431.38836	7.50
35	α-Tocopherol-*d6*	Pos	437.42602	7.50
36	Vitamin D_3	Pos	385.34649	6.70
37	Vitamin D_3-*d3*	Pos	388.36532	6.70
